# Environmental Epigenetics of Diesel Particulate Matter Toxicogenomics

**DOI:** 10.3390/ijerph17207386

**Published:** 2020-10-10

**Authors:** Stephanie M. Bilinovich, Kristy Lewis, Barbara L. Thompson, Jeremy W. Prokop, Daniel B. Campbell

**Affiliations:** 1 Department of Pediatrics & Human Development, Michigan State University, Grand Rapids, MI 49503, USA; bilinovi@msu.edu (S.M.B.); lewisk50@msu.edu (K.L.); thom1756@msu.edu (B.L.T.); prokopje@msu.edu (J.W.P.); 2 Center for Research in Autism, Intellectual, and other Neurodevelopmental Disabilities, Michigan State University, East Lansing, MI 48824, USA; 3 Neuroscience Program, Michigan State University, East Lansing, MI 48824, USA; 4 Department of Pharmacology and Toxicology, Michigan State University, East Lansing, MI 48824, USA

**Keywords:** autism spectrum disorder, diesel particulate matter, epigenetics, toxicogenomics

## Abstract

Autism spectrum disorder (ASD) is a neurodevelopmental disorder characterized by disruptions in social communication and behavioral flexibility. Both genetic and environmental factors contribute to ASD risk. Epidemiologic studies indicate that roadway vehicle exhaust and in utero exposure to diesel particulate matter (DPM) are associated with ASD. Using the Comparative Toxicogenomics Database (CTD), we identified genes connected to DPM exposure and ASD, extracted the known enhancers/promoters of the identified genes, and integrated this with Assay for Transposase Accessible Chromatin (ATAC-seq) data from DPM-exposed human neural progenitor cells. Enhancer/promoter elements with significantly different chromosome accessibility revealed enriched DNA sequence motifs with transcription factor binding sites for EGR1. Variant extraction for linkage disequilibrium blocks of these regions followed by analysis through Genome Wide Association Studies (GWAS) revealed multiple neurological trait associations including exploratory eye movement and brain volume measurement. This approach highlights the effects of pollution on the regulatory regions of genes implicated in ASD by genetic studies, indicating convergence of genetic and environmental factors on molecular networks that contribute to ASD. Integration of publicly available data from the CTD, cell culture exposure studies, and phenotypic genetics synergize extensive evidence of chemical exposures on gene regulation for altered brain development.

## 1. Introduction

There has long been a known relationship between the environment and human health. Exposures to environmental contaminants have been linked to diseases including cancer [1], respiratory illnesses [2], and neurodevelopmental disorders [3]. A growing body of research has supported the hypothesis that environmental conditions, like malnutrition, chemical, and viral exposures during in utero development can have negative effects on both adult health and early childhood health [4]. Air pollution is a ubiquitous environmental contaminant that impacts human health. Air pollution from vehicle exhaust is a complex mixture of organic chemicals, particulate matter, and volatile gases. Diesel particulate matter (DPM) is a specific component of air pollution that originates from truck exhaust and has been linked to adverse health consequences including lung cancers, asthma, cardiovascular disease [5], low birth weight [6], and autism spectrum disorders (ASD) [7]. The exact mechanisms by which DPM impacts human health is likely a complex mechanism, with growing evidence that DPM exposure contributes to oxidative stress [8] and inflammation [9]. 

Given the evidence environmental toxins impact human health, it is vital that a public database curates the experimental data reporting these effects. The Comparative Toxicogenomics Database (CTD) is a public database that aims to advance understanding of environmental exposure impact on human health [10]. The CTD contains information on the interactions between genes, diseases, and chemicals [10]. The CTD is a useful resource for examining previously published evidence on the effects of environmental toxins, providing insights into the chemical, gene, and disease interactions. Resources such as the CTD provide a database for the development of hypothesis generation and testing using extensive genomic technologies, from gene regulation to genomic variant extraction. Here, we generated hypotheses using publicly curated data in the CTD on the role of air pollution on the expression of genes involved in autistic disorder and examined these alterations to gene expression with ATAC-seq data we generated in human neural progenitor cells exposed to an increasing dose of diesel particulate matter (DPM). ATAC-seq (Assay for Transposase-Accessible Chromatin using sequencing) is used to identify chromatin accessibility genome-wide [11]. The technique involves the use of transposon insertions in regions where the chromatin is open and accessible, allowing for subsequent sequencing of the region. Any regions in the DNA where the chromatin is closed will prevent sequencing. The ability of ATAC-seq to identify regions of open chromatin makes it an ideal technique for examining epigenetic mechanisms of gene expression. ATAC-seq has been widely used to probe biological questions of gene regulation including normal human tissues [12], differences between cancerous and healthy cells [13], effects of therapeutics on cells [14], nucleosome mapping [15], enhancer identification [16], and transcription factor binding sites [17]. This technique was used here to examine the effects of DPM on chromatin accessibility at the enhancers for genes identified by the CTD as related to both ASD and susceptible to vehicle exhaust particulate matter. 

## 2. Materials and Methods 

### 2.1. Cell Culture

ReNcell CX human neural progenitor cells (Sigma Millepore #SCC007) were maintained in tissue culture flasks at 37 °C and 5% CO_2_ in ReNcell NSC Maintenance Medium supplemented with FGF and EGF in laminin-coated flasks. Diesel particulate matter was purchased from Sigma, serially diluted in a small volume of dimethyl sulfoxide (Sigma-Aldrich #NIST2975) and added to the ReNcell CX medium for 24 hours prior to harvest. A total of 50,000 cells from each dose (*n* = 2) were used for ATAC-seq protocol as described previously [11]. Fragmented DNA libraries were prepared using the Illumina Nextera Index Kit and adaptors (cat no. # FC-121-1030, FC-121-1011). Sequencing was performed by the Van Andel Institute Genomics Core, using Illumina NextSeq 500, paired end sequencing with all samples run over two flow cells.

### 2.2. ATAC-Seq Analysis 

ATAC-seq analysis was performed using the ENCODE ATAC-seq pipeline [18] where same sample replicates from the two flow cells were concatenated and aligned against the hg38 index. Pipeline default parameters were used with the exception of bowtie2, set for very sensitive and maximum insert size at 1000 bp [19]. MACS2 output files were filtered of mitochondrial reads, PCR duplicates and blacklisted regions. 

### 2.3. Database Analysis

The genes associated with both vehicle exhaust and particulate matter were downloaded from the CTDbase (data accessed July 2020). Data was filtered for genes that occur in both datasets and then further filtered to extract out the genes that are curated into the database as being associated with autism spectrum disorder (ASD). The UCSC genome browser [20], using the GRCh38/hg38 genome build, was used with GeneHancer [21] regulatory elements to find the enhancers associated with the ASD associated genes identified in the CTDbase. 

### 2.4. ATAC-Seq Differential Accessibility Analysis and Visualization

The sequences from these 772 enhancer regions of 78 ASD DPM genes from the CTD were extended 300 bp upstream and downstream of the start and end positions using the GenomicRanges R package. Differential Accessibility (DA) analysis of just these regions was performed using CSAW following recommended methodology by the developers [22] using the loess normalization and MACS2 peaks [23]. Batch effects were accounted for using the edgeR package in the design matrix using model.matrix according to methodology recommended [24]. Pairwise DA was used for each concentration of DPM using control DPM0 as baseline. DPM10 had 60 regions of significant DA (Pval < 0.05), DPM20 had 64 significant DA regions, DPM50 had 76 significant DA regions and DPM100 had 156 significant DA regions. Annotation of regions was performed by the ChIPseeker and bioMaRt R packages to the nearest promoter region. Peaks were visualized using Integrative Genomics Viewer [23] using generated bigwig fold change signal files from MACS2 [23]. The gene enhancer regions that had an FDR < 0.05 and a log2FC compared to the control cells of <−1.0 or >1 were split to the negative and positive genes and the sequences were submitted to the MEME [25] database and the identified motifs were submitted to TOMTOM [26] to identify the transcription factors that bind to those motifs, with the JASPAR [27] redundant vertebrates 2018 option. STRING [28] analysis and an interactome of the genome-wide ASD associated genes [29] were performed on the identified genes with significant enhancer regions and transcription factors.

### 2.5. Genomic Variant Extraction

The 80 significant regulation regions identified were parsed through gnomADv3 [30] for any genomic variants in a diverse population of 71,702 whole genomes. Variants were filtered through total allele frequency greater than 0.0002. Those variants with an rsID were parsed through linkage disequilibrium (LD) using SNiPA [31] within any of the 1000 genome phase 3 v5 populations, yielding a total list of variants connected in inheritance with >0.8R^2^ to those variants within the enhancers. The genome-wide association studies GWAS catalog represents a highly curated list of variants to traits that reach genome wide significance, as reviewed by an expert panel of geneticists. The GWAS catalog [32] was obtained on 8/23/2020 and parsed for any SNPs from the LD list, searching rsIDs in LD to enhancers against the rsIDs within the GWAS catalog. From this, the connected traits to matched rsIDs from the GWAS catalog were reviewed for any that have neurological function. 

## 3. Results

**Figure 1:**
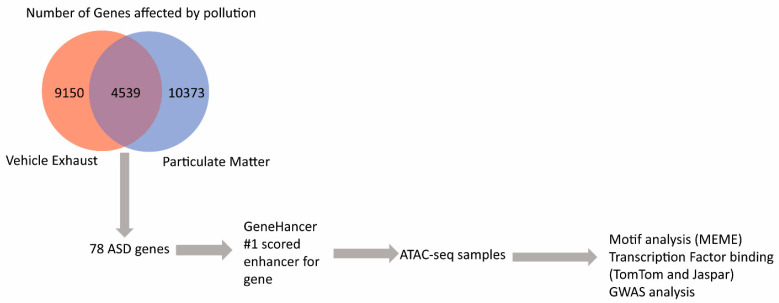
Flow chart of analysis. Data from the publicly available comparative toxicogenomic database (CTD) identified 4539 genes that were differentially expressed following exposure to both vehicle exhaust and particulate matter. Genetic studies have implicated 78 of those genes in autism spectrum disorder (ASD). Enhancer regions for these 78 genes were identified and analyzed for differential accessibility by ATAC-seq on DPM-exposed human neural progenitor cells. Motif analyses of the open chromatin identified transcription factor binding sequences.

To examine the role of diesel particulate matter (DPM) on gene expression, we used a multifaced approach by combining gene expression data about air pollution and DPM from the CTD, linking of enhancers to those differentially expressed genes using GeneHancer, analysis of the identified enhancers by ATAC-seq of DPM-exposed neural progenitor cells, and genomic variant phenotype associations from GWAS (Figure 1). We used two search terms in CTD to identify the genes that are affected by two forms of pollution, vehicle emissions and particulate matter. For both vehicle emissions and particulate matter, of the top 20 biological pathways affected by the pollutant, three were relevant to ASD molecular biology: gene expression (#6), developmental biology (#9), and axon guidance (#17). Both pollutants are correlated with ASD on CTD, ranking 2nd for vehicle emissions and 28th for particulate matter. There are a total of 9150 genes associated with vehicle exhaust and 10,373 genes associated with particulate matter. We took the overlapping 4530 genes and then extracted the genes associated with ASD [33,34], resulting in a total of 78 genes. These ASD genes in the CTD are curated from the literature and the Online Mendelian Inheritance in Man (OMIM) database. Of these genes labeled as associated with ASD in the CTD, 42 are considered ASD risk genes in the Simons Foundation Autism Research Initiative (SFARI) database [35] and eight of the genes were found to be significant in a recent exome sequencing of ASD [36].

**Figure 2:**
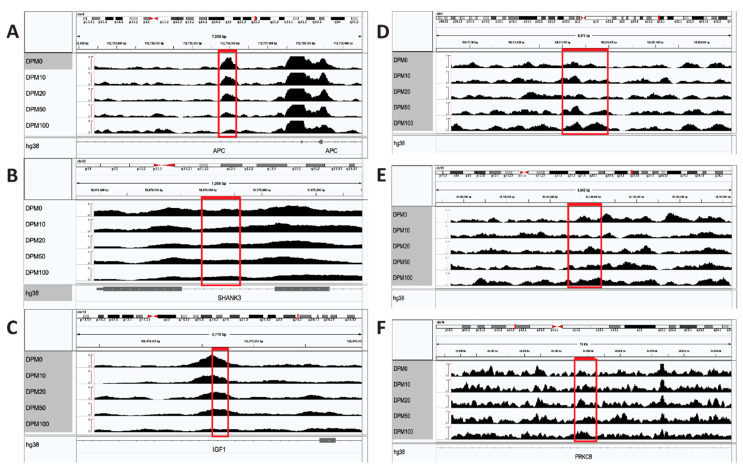
Top gene enhancers that are differentially accessible when exposed to increasing doses of diesel particulate matter (DPM). (**A**)–(**C**): three enhancer regions that have negative log2FC in chromatin accessibility compared to the control following increasing DPM exposure, and are associated with genes *APC* (**A**), *SHANK3* (**B**), and *IGF1* (**C**). (**D**)–(**F**): the enhancer regions with positive log2FC compared to the control following increasing DPM exposure, and are associated with the genes *PTGS2* (**D**), *PTEN* (**E**), and *PRKCB* (**F**). Both the enhancers for *PTGS2* (**D**) and *PTEN* (**E**) are located in the distal intergenic region.

**Figure 3:**
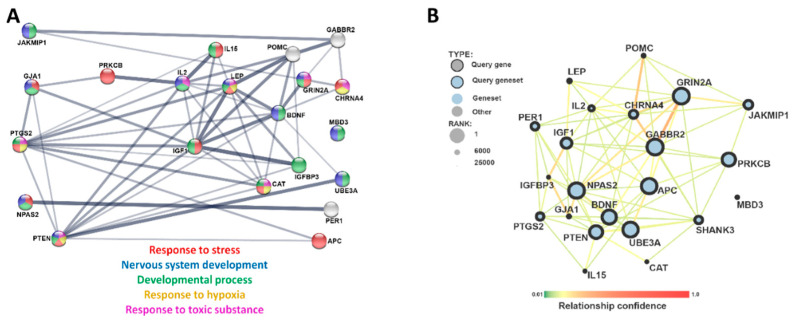
STRING analysis of the genes associated with the enhancers of interest. (**A**) The 4 genes on the far left are the genes with enhancer regions that are more accessible following DPM exposure and the 17 on the right enhancer regions that are less accessible following DPM exposure. The single gene that has both negative and positive enhancer regions, *PRKCB*, is highlighted in the red box in the middle of the plot. The gene *SHANK3* is not included on this chart because it is not in the STRING human protein database. Gene ontology enrichment terms are highlighted by color. There is an enrichment for the following biological processes based on gene ontology: response to stress (FDR 0.00041), nervous system development (FDR 0.00046), developmental process (FDR 0.00046), response to hypoxia (FDR 0.00021), and response to toxic substance (FDR 1.84 × 10^−5^). (**B**) Interaction map of the 22 genes with enhancers that are differentially accessible visualized using an interaction network of genome wide ASD genes.

For these 78 genes, GeneHancer [20] was used to find all the associated regulatory regions (enhancers/promoters). A total of 772 regulatory regions were examined in ATAC-seq data on ReNcell CX neural progenitor cells exposed to five concentrations of DPM (0, 10, 20, 50, 100 µg/mL). Of these 772 regulatory regions, 641 were found in the ATAC-seq dataset, with 80 of the elements having an FDR less than 0.05 at the 100 µg/ml DPM dose relative to the control. Further parsing of the data using a cut off less than −1.0 log2 fold change (FC) or greater than +1.0 log2FC resulted in 22 negative regions of interest and seven positive regions (Table S1). Out of the 29 combined regions, 22 are uniquely associated to genes, with only *PRKCB* having multiple regulatory regions (both negative and positive). The genes with the highest positive log2FC regulatory regions include *PTGS2*, *PTEN*, and *PRKCB* (Figure 2). The genes with the biggest negative log2FC regulatory regions were *APC*, *SHANK3*, and *IGF1* (Figure 2). A STRING plot of the 22 genes shows that many of the genes are associated with each other and have overlapping biological functions (Figure 3). These biological processes include nervous system development and response to stress. This overlap of the genes with altered enhancer regions shows the DPM exposure is likely affecting these regulatory regions of the genes which are enriched in response to stress (i.e., hypoxia and toxic substances exposures) and developmental processes.

**Figure 4:**
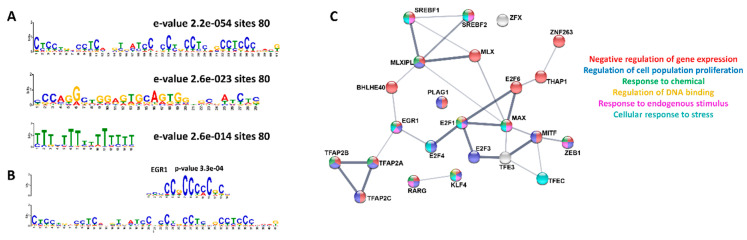
MEME motif analysis of the regulatory regions. (**A**) shows the 3 MEME motifs found for the 80 GeneHancer regions (FDR < 0.05) associated with the ASD curated genes in the CTD. (**B**) EGR1 is the top transcription factor that recognizes the top identified motif based on TomTom and JASPAR database search. (**C**) STRING plot of the transcription factors that recognize the top MEME motif. Colors correspond to the biological processes’ enriched terms based on gene ontology in the network. The biological processes found to be enriched include: negative regulation of gene expression (FDR 4.30 × 10^−15^), regulation of cell population proliferation (FDR 1.17 × 10^−7^), response to chemical (FDR 0.0289), regulation of DNA binding (FDR 0.0404), response to endogenous stimulus (FDR 0.0015), and cellular response to stress (0.0031).

Since these genes themselves are involved in similar biological processes during development and response to stress, it is possible that DPM alters transcription through overlapping intracellular mechanisms. Similar recognition sequences within the elements regulated by the same transcription factors may be activated by DPM. Using the MEME-suite [25], we identified redundant repetitive sequences (DNA motifs) in the 80 regulatory regions (FDR < 0.05), resulting in motifs that are repeatedly found in the regulatory regions. The three motifs (Figure 4A) all have significant e-values of less than 1 × 10^−14^. The motifs were assessed through TomTom using the JASPER database to match motifs to transcription factor recognition, identifying 29 unique transcription factors that map to the top MEME motif result. The top transcription factor results for the 1st MEME motif is *EGR1* (Figure 4B). Egr1, or the early growth response protein, is regulated in oxidative stress [37], and the *EGR1* number 1 curated chemical interaction is with particulate matter in the CTD [38]. When we compared the 29 unique transcription factors using a STRING plot, we found an enrichment in genes related to the biological functions: gene expression, cell population proliferation, response to chemicals and stress, and regulation of DNA binding (Figure 4C). As both the 22 genes with altered enhancer regions and the transcription factors that target these enhancer rights have shared biological processes of responses to stress and general cellular processes like cell proliferation and developmental process, it shows that DPM exposure is targeting specific biological functions. It has previously been shown that DPM exposure causes oxidative stress [39,40]. This gives possible insight into how DPM exposure affects gene regulation by targeting the enhancer regions of genes and their transcription factors, which can affect expression of the genes that are critical for development.

**Figure 5:**
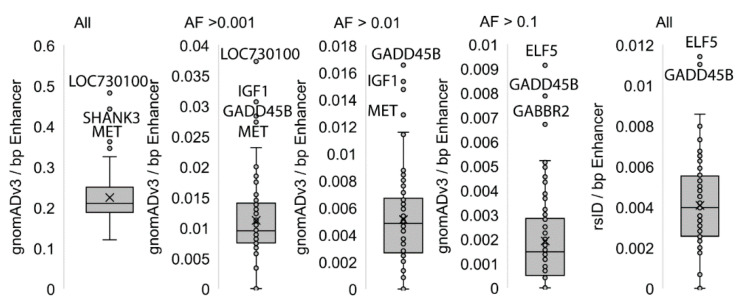
Genomic variants found within top regions. The first four box and whisker plots are based on gnomADv3 whole genome extraction and the last based on all rsID listed variants. All values are the number of variants mapped per base pairs (bp) of element. Outliers are labeled based on GeneHancer connected gene. The four gnomADv3 plots show all variants (far left) followed by cutoffs for allele frequency within all populations (cutoffs for 0.001, 0.01, and 0.1).

To test the impact on neuronal development for these identified regulatory regions, we extracted genomic variants linked to any region with FDR significance. This analysis is an independent observation of the identified regulatory regions connected to neurological phenotypes, with no gene level ontology biases for connection of neurological function. Using the gnomADv3 database of 71,702 whole genomes, we identify 60,186 unique variants within the regulatory regions, with 3250 > 0.001, 1561 > 0.01, and 593 variants > 0.1 allele frequency within the population. Normalizing the number of variants per number of bases within each regulatory region, we identified elements associated with *LOC730100* and *SHANK3* for all variants to have high SNP density, with marked density of high allele frequency variants within elements for *GADD45B*, *IGF1*, *MET*, *ELF5*, and *GABBR2* (Figure 5). Of these variants, 1207 have rsID numbers and are connected to 10,659 SNPs based on 0.8 R^2^ linkage disequilibrium from all populations, imputing all SNPs that are coinherited with SNPs within the regulatory regions identified. Assessing all of the SNPs through the GWAS catalog [32] (196,813 associations from 4671 publications) identified 120 associations, with 12 (10%) of them linked to neurological phenotypes (Table 1) including self-reported educational attainment, multiple sclerosis, exploratory eye movement measurement, schizophrenia, risk-taking behavior, and brain volume measurement. The most interesting observation was rs703545, which is associated with brain volume measurement (*p* = 1.00 × 10^−8^). rs703545 has a 0.9 R^2^ to rs2114912, 0.86 to rs2607988, and 0.85 to rs2245763, all of which are found within enhancers for *IGF1*. These SNPs are present within all populations based on the single nucleotide variant 12-102943000-A-G(CRCh37) in v2.1.1 in gnomAD [30]. *IGF1* is a high-confidence ASD gene [35], which has significant decreasing accessibility following DPM treatment (Figure 2C). Overall, this suggests that regulatory region integrated mapping for DPM based on the CTD and ATAC-seq of exposure has neurological phenotype connections.

## 4. Discussion

The epigenetic effects of environmental exposures impact human development and health. Publicly available databases like the CTD are important tools in evaluating the effects of chemicals on not only the genome but also the epigenome. The curated CTD contains information on 2,159,899 chemical–gene interactions (13,618 unique chemicals) and 27,723,024 gene–disease associations as of July 2020 [10]. As presented here, this data can be a strong tool in evaluating the role of the environment on health. By building onto the knowledge of previous experiments using ATAC-seq data from DPM-exposed progenitor cells, new insights into how environmental chemical exposures affect human health can be identified.

A closer examination of the top three genes with enhancers that are less accessible (*APC*, *SHANK3*, and *IGF1)* shows that genes associated with DPM response cause changes to gene expression or methylation states. *SHANK3* was found to have a reduced mRNA expression levels in early postnatal rats exposed to DPM [41]. The *APC* gene is hypermethylated in cells following DPM exposure [42]. In addition to the less accessible enhancers, there are also alterations to *PTGS2* [43], *PTEN* [9,44], *PRKCB* [45] expression upon exposure to DPM. We have not only found enhancers for genes that are related to processing this stress, but also those regions of the genome that have similar recognition sequences that also attract transcription factors related to the processing of toxic substances. Some of these transcription factors are known to have shared biological functions. In the case of *PTEN* regulation, the transcription factor found to target the motif in the *PTEN* enhancer region is a target for the EGR1 has been shown to control expression [46].

*EGR1* (NGFI-A, zif268) is an immediate early gene and inducible transcription factor. Within the CTD, 1236 gene–chemical interactions are identified [38]. The top chemical interactor for *EGR1* is particulate matter with 43 curated interactions. *EGR1* has been shown to have altered gene expression in the response to environmental pollutants like arsenic [47], bisphenol A, and lead [48]. Exposure of bronchial epithelial cells to diesel particulate matter has been shown to decrease expression of EGR1 [49]. It has been shown to be involved in pulmonary inflammation following particulate matter exposure [50], increased expression by polycyclic aromatic hydrocarbons and 2,3,7,8-tetrachlorodibenzo-p-dioxin exposures [51] and increased mRNA levels have been found in microvascular endothelial cells following ultrafine particle and cigarette smoke extract exposures [52]. Additionally, *Egr-1* expression is disrupted by neonatal ethanol exposure [53], is differentially impacted by age and experience [54], and can be induced by both anxiogenic and anxiolytic drugs, indicating a sensitivity of Egr-1 to physiological homeostasis [55]. The function of EGR1 in neurodevelopment includes regulation of synaptic plasticity [56] and maturation of neurons in the dentate gyrus [57]. For an extensive review of established function of EGR-1 in neurodevelopmental disorders and upstream and downstream targets, see Duclot and Kabbaj [58].

Of the 78 genes that were curated as having an association with ASD in the CTD, 41 are also in the SFARI database. While most of these genes curated to be associated with ASD in the CTD are the results of rare single gene mutations, many also are the result of associations of common genetic variants to ASD. Of the 29 enhancers identified here as affected by DPM exposure, they are associated with 22 specific genes, 11 of which are considered ASD risk genes [35] (three high confidence, two strong candidate, and six suggestive evidence genes). These three high confidence genes are *SHANK3*, *UBE3A*, and *PTEN*, with both *UBE3A* and *PTEN* having more than one enhancer affected by DPM exposure. GWAS studies demonstrated that exposures to air pollution alter gene expression of ASD genes *SHANK3* [41], *GADD45B* [59], and *IGF1* [60].

Many ASD risk genes are the result of genetic mutation. But sequencing studies have shown that there are loss-of-function variants for genes in unaffected individuals. These mutations may be exacerbated by environmental factors and this interaction contributes to disease risk. One key example of this is the *MET* gene. Studies have shown that individuals with risk variants in the *MET* promoter combined with high exposures to traffic related pollution have an increased risk of ASD [61]. Additionally, *PRKCB* gene variants have decreased gene expression in individuals with a diagnosis of ASD [62], and also show decreased expression following exposures to particulate matter [63]. These two examples of genetic variants that can also be altered by an environmental agent indicates that there are likely epigenetic mechanisms that contribute to disease state when combined. Additionally, there is a well-established role of the paternal age to incidence of ASD in offspring, where rare genetic changes due to age and environmental exposures of sperm increase genes identified in ASD and other neurodevelopmental disorders [64]. Many studies have thus established that not only rare variants contribute to ASD, but that there is a complex interplay of environmental exposures with genetics to contribute to elevated risk of ASD [65,66,67]. The role of environmental exposures on neurodevelopment in ASD can be extended to other neurodevelopmental and psychiatric disorders. The ASD risk genes are commonly key components of basic neurodevelopment and are also frequently implicated in other neurodevelopmental and psychiatric disorders such as schizophrenia [68], intellectual disability [69], and epilepsy [70].

There are some limitations to the study. The CTD can help to give insights into the relationship between genes and toxins, but care should be taken to examine the gene–disease relationship critically. The CTD is a useful tool for hypothesis generation, but it is susceptible to the same candidate gene biases as other databases. Of the 78 genes related to ASD based on their curation in the CTD, there was little overlap with databases like SFARI [35] and other publications [36], indicating discrepancies between the list of ASD risk genes. The CTD genes associated with ASD and particulate matter were used for consistency throughout the analysis, which influences the enhancer regions used. It is possible that using ASD associated genes from other sources may yield different enhancers. In addition to a bias from the database, it is possible that the enhancer regions identified could be modulating gene expression for multiple genes [71].

While this work was focused on vehicle exhaust/particulate, this workflow can be applied to studying the toxicogenomic mechanisms of other diseases and pollutants. Our results clearly build onto previously curated data that indicates vehicle exhaust/particulate matter can have wide ranging effects to gene expression and regulation. By examining chromatin accessibility, we gained insights into possible mechanisms of DPM exposure on development. DPM exposure has been previously linked to alterations in oxidative stress [72], brain development [73], and inflammation [74]. We have shown here that gene enhancers that regulate ASD related genes are also altered following DPM exposure. These alterations to the enhancers could provide much needed insights into how DPM affects gene expression, and how these alterations in gene expression could drive altered neurobiology during fetal brain development.

## 5. Conclusions

Exposure to diesel particulate matter affected the chromatin accessibility of autism risk genes. Transcription factor binding sites in these regulatory regions are affected by DPM exposures likely affecting gene expression.

## Figures and Tables

**Table 1:** Neurological linked GWAS to regulated enhancers. All SNPs found within identified enhancers in addition to all population linked 0.8R^2^ SNPs were queried within the GWAS catalog obtained on8/23/2020. In red text is a SNP linked to the highlighted region in Figure 2C. CHR = chromosome, POS = position.

Linked Regulatory Region	PubMed_ID	MAPPED_TRAIT	RISK ALLELE	CHR_ID	CHR_POS	*p*-Valume
chr2:51031736-51032889	30038396	self-reported educational attainment	rs12620796-A	2	51060711	9.00 × 10^−11^
chr5:148730602-148971200	30287806	nicotine dependence symptom count, depressive symptom measurement	rs57108954-T	5	148857822	3.00 × 10^−6^
chr5:148730602-148971200	26242244	exploratory eye movement measurement	rs17108911-?	5	148903759	6.00 × 10^−6^
chr7:22600600-22602886	29071344	unipolar depression, alcohol dependence	rs2905347-G	7	22580700	6.00 × 10^−6^
chr7:116685939-116778343	19010793	multiple sclerosis	rs10243024-?	7	116706549	6.00 × 10^−6^
chr8:117846474-117850990	30038396	mathematical ability	rs17430287-A	8	117844068	4.00 × 10^−8^
chr9:4506589-4512250	29503163	schizophrenia, response to risperidone	rs16921385-A	9	4507513	4.00 × 10^−8^
chr10:88060002-88390829	31604244	multiple sclerosis	rs1819577-A	10	88067364	1.00 × 10^−7^
chr10:88060002-88390829	30038396	self-reported educational attainment	rs1426619-T	10	88331783	1.00 × 10^−11^
chr10:88060002-88390829	30038396	self-reported educational attainment	rs1426619-T	10	88331783	1.00 × 10^−10^
chr11:27720334-28567694	30643258	risk-taking behavior	rs16918024-T	11	28566879	8.00 × 10^−9^
chr12:102474603-102536836	31676860	brain volume measurement	rs703545-?	12	102549222	1.00 × 10^−8^

## Supplementary Materials

The following are available online at https://www.mdpi.com/1660-4601/17/20/7386/s1, Table S1: List of enhancer regions.
